# Computational Analysis of mRNA Expression Profiles Identifies the *ITG* Family and *PIK3R3* as Crucial Genes for Regulating Triple Negative Breast Cancer Cell Migration

**DOI:** 10.1155/2014/536591

**Published:** 2014-05-06

**Authors:** Sukhontip Klahan, Mei-Shin Wu, Edward Hsi, Chi-Cheng Huang, Ming-Feng Hou, Wei-Chiao Chang

**Affiliations:** ^1^Department of Clinical Pharmacy, Taipei Medical University, Taipei 110, Taiwan; ^2^Department of Medical Research, Kaohsiung Medical University Hospital, Kaohsiung 807, Taiwan; ^3^Graduate Institute of Biomedical Electronics and Bioinformatics, National Taiwan University, Taipei 106, Taiwan; ^4^Cathay General Hospital SiJhih, New Taipei 221, Taiwan; ^5^School of Medicine, Fu-Jen Catholic University, New Taipei 242, Taiwan; ^6^School of Medicine, Taipei Medical University, Taipei 110, Taiwan; ^7^Cancer Center, Kaohsiung Medical University Hospital, Kaohsiung 807, Taiwan; ^8^Department of Surgery, Kaohsiung Medical University Hospital, Kaohsiung 807, Taiwan; ^9^Kaohsiung Municipal Ta-Tung Hospital, Kaohsiung 801, Taiwan; ^10^Department of Pharmacy, Taipei Medical University-Wan Fang Hospital, Taipei 116, Taiwan; ^11^Master Program for Clinical Pharmacogenomics and Pharmacoproteomics, School of Pharmacy, Taipei Medical University, Taipei 110, Taiwan

## Abstract

Triple-negative breast cancer (TNBC) is an aggressive type of breast cancer that does not express estrogen receptor (ER), progesterone receptor (PR), and human epidermal growth factor receptor (Her2/neu). TNBC has worse clinical outcomes than other breast cancer subtypes. However, the key molecules and mechanisms of TNBC migration remain unclear. In this study, we compared two normalized microarray datasets from GEO database between Asian (GSE33926) and non-Asian populations (GSE46581) to determine the molecules and common pathways in TNBC migration. We demonstrated that 16 genes in non-Asian samples and 9 genes in Asian samples are related to TNBC migration. In addition, our analytic results showed that 4 genes, *PIK3R3, ITGB1, ITGAL*, and *ITGA6*, were involved in the regulation of actin cytoskeleton. Our results indicated potential genes that link to TNBC migration. This study may help identify novel therapeutic targets for drug development in cancer therapy.

## 1. Introduction

Breast cancer is the second most common cancer worldwide with an estimated 1.38 million incident cases in 2008 [1]. Triple-negative breast cancer (TNBC) is a highly aggressive type of breast cancer and refers to tumors that lack expressions of estrogen receptor (ER), progesterone receptor (PR), and human epidermal growth factor receptor 2 (HER2) genes [2]. In 2012, approximate 15%~20% of all breast cancer cases were diagnosed as TNBC [3], which had limited or ineffective treatment options. High risks of visceral metastasis [4], worse prognosis [5], and frequent relapses [6] make TNBC a major issue for global public health.

Dent et al. [7] showed that women with TNBC had an increased likelihood of distant recurrence within 5 years of diagnosis (hazard ratio: 2.6) compared to non-TNBC cases. Another study found that women with TNBC were more likely to experience a visceral metastasis within 5 years of diagnosis than other types of cancer (hazard ratio: 4.0) [8]. Moreover, several reports showed that TNBC has a shorter distant metastasis-free survival [9, 10] compared to hormone-sensitive or HER2-positive breast cancer. According to the Surveillance, Epidemiology, and End Results (SEER) database the incidence rate, mortality rate, and five-year survival rate of TNBC are varied between different populations [11]. Previous study also showed that TNBC subtype was more frequent in African-Americans compared with Caucasians (adjusted odd ratio: 1.98) [12]. In addition, TNBC has the worst clinical pattern among all subtypes of breast cancer [13, 14]. Cell migration is a key factor in many events of cancer metastasis [15, 16]. In this study, we aimed to identify crucial molecules or common pathways which are involved in the metastasis of TNBC. Thus, the combination of two normalized microarray datasets from GEO database was used, with one dataset including microarray data from 41 non-Asian TNBC samples [17] and another dataset containing microarray data from 26 Taiwanese TNBC samples [18]. Differences in gene expressions between the early and late stages were calculated by Significance Analysis of Microarrays (SAM), and the pathway analysis was carried out using the Ingenuity Pathway Analysis (IPA).

## 2. Methods and Materials

### 2.1. Data Resources

The Gene Expression Omnibus (GEO) is a public repository in which large numbers of high-throughput sequencing data and microarray datasets are stored. We used the keyword “triple negative breast cancer” to search for the material for our study from the GEO repository and found 683 qualified datasets. We then further filtered out datasets which were uploaded before 2012 and excluded in vitro studies and then obtained 353 datasets after the filtering process. Among the 353 datasets, both studies of Lindner et al. [17] and Kuo et al. [18] provided comprehensive information of patients' pathological stage of TNBC as well as sufficient sample sizes for our classification rule and matched our goal to search for common pathways on different races. These two datasets were deposited in the GEO database under the respective codes GSE46581 and GSE33926. Data of GSE46581 were generated by Illumina HumanRef-8 WG-DASL v3.0 (GPL8432), a Whole-Genome DASL Assay which simultaneously analyzes over 24,000 genes with high sensitivity [19]. Data of GSE33926 were generated from an Agilent-012097 Human 1A Microarray (V2) (GPL7264), a platform that contains over 20,000 oligonucleotide probes to detect gene expressions; this platform was reported to generate data with high confidence [20].

### 2.2. Data Processing

Lindner et al. (GSE46581) recruited 90 TNBC patients, profiled gene expressions and immunohistochemistry, and further investigated differences between European-American and African-American patients at a molecular level. Among the 90 samples, we selected 25 pathological stage I breast cancer patients as well as 16 patients at pathological stages III and IV and compared the total gene expression between these two groups. The project by Kuo et al. (GSE33926) recruited 51 TNBC Taiwanese patients and determined 45 signature genes for predicting TNBC metastasis. In the GSE33926 dataset, there were 12 pathological stage I patients and 14 patients at pathological stages III and IV. For both datasets, patients at pathological stage I without sign of cancer migration or metastasis found in regional lymph nodes and pathological stages III and IV patients with signs of migration or development of cancer cells in regional lymph nodes were classified as early-stage and late-stage groups, respectively, according to the American Joint Committee on Cancer (AJCC) TNM system [21]. In addition, pathological stage II patients were excluded due to unclear lymph node involvement. For both datasets, we downloaded normalized data. Gene expressions of GSE33926 were normalized using quantile normalization as described in their article [18]. Both SAM and IPA were applied to identify the common pathways for TNBC.

### 2.3. Gene Expression Calculations

Gene expression profile calculations were conducted using SAM method [22] and R (vers. 2.15.1). For each dataset, we applied SAM to calculate and compare expression differences between early- and late-stage cohorts. Differentially expressed genes between early- and late-stage groups were identified using a 2-sided Student's *t*-test (*P* < 0.05) and comparisons of multiples of change (>2-fold). The percentage of these significant genes identified by chance was referred to the false discovery rate (FDR). SAM provided the *q* value as the score of FDR for each significant gene.

### 2.4. Pathway Analysis

The IPA (Ingenuity Systems, Mountain View, CA, USA; available at www.ingenuity.com) is a literature-based program for pathway analysis and gene function annotation. The gene network analysis is to cluster the genes based on their molecular functions and present their correlations. We applied the IPA to explore gene networks and mine the pathways which might be involved in cell migration and tumor metastasis of TNBC. We used the keyword “migration” to search the IPA database and selected networks related to cancer cell migration. For each dataset, the up- and down-regulated genes were mapped to the selected networks. We further compared the IPA mapping results between the two GEO datasets (GSE46581 and GSE33926) and determined the common molecules or pathways which had significant effects on TNBC cell migration.

### 2.5. DAVID Network Analysis

DAVID online database [23] provides a comprehensive set of functional annotation tools to visualize the pathways of our interested genes. After IPA analysis process, we selected 5 genes from GSE46581 and 2 genes from GSE33926 to further search for a common pathway of TNBC in DAVID database.

## 3. Results

### 3.1. Clinical Characteristics and Gene Expressions

The criteria to determine pathological cancer stages include the tumor size, lymph nodes status, and metastasis status. Stages I and III/IV have clear definitions of cancer of whether it has reached nearby lymph nodes or not. However, the lymph node status is heterogenic in stage II. To avoid any ambiguity, we excluded patients who were classified as pathological cancer stage II. As shown in Tables 1 and 2, there was no significant difference in age between the two groups of the two datasets. The SAM program calculated gene expressions changes between pathological stage I and stages III/IV patients. In the American dataset (GSE46581), 18,345 out of 24,000 target genes were analyzed, and 433 upregulated genes and 241 downregulated genes were found to reach statistical significance. In the Taiwanese dataset (GSE33926), 20,140 genes were analyzed, and 48 upregulated genes and 77 downregulated genes were observed to have statistically significant difference between early- and late-stage groups.

### 3.2. Common Genes between Asians and Non-Asians

After the gene expression calculation process, we found 5 genes with significant expression changes that commonly existed in both Asian and non-Asian samples. Among the 5 genes,* ACTA1, C4orf7, CYP26B1*, and* PRAME* were downregulated in both Asian and non-Asian TNBC populations, while* ASPN* was the only gene that was upregulated in both populations (Table 3).

### 3.3. Pathway Analysis of TNBC from Non-Asian and Asian Patients

IPA was applied to annotate the cell migration-related genes and the networks. Patients' ethnic origins in the GSE46581 dataset were European and African-American. In the non-Asian samples (GSE46581), there were 16 genes with significant expression changes related to cell migration, whereas 9 cell migration-related genes were found in the Asian samples (GSE33926) (Table 4). However, no common cell migration-related gene was identified in both these two populations.

**Figure 1:**
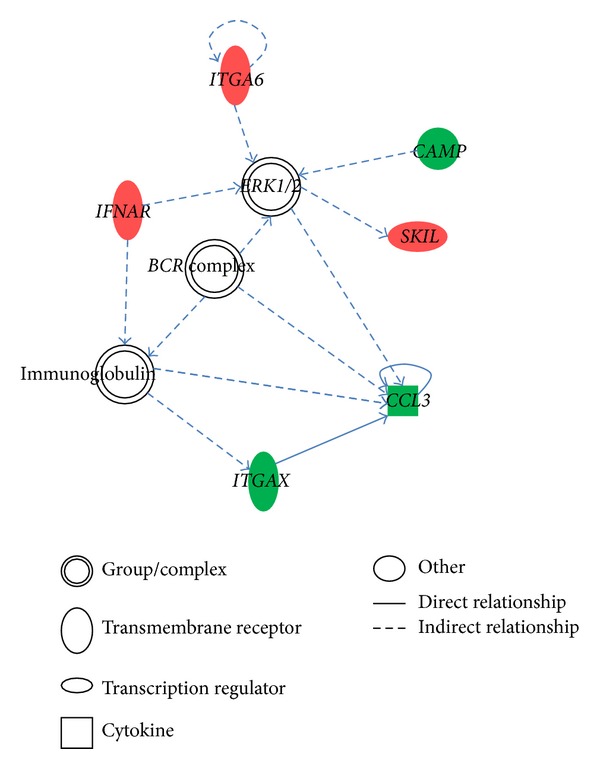
Cell death and survival, developmental disorders, skeletal and muscular disorders network.

**Figure 2:**
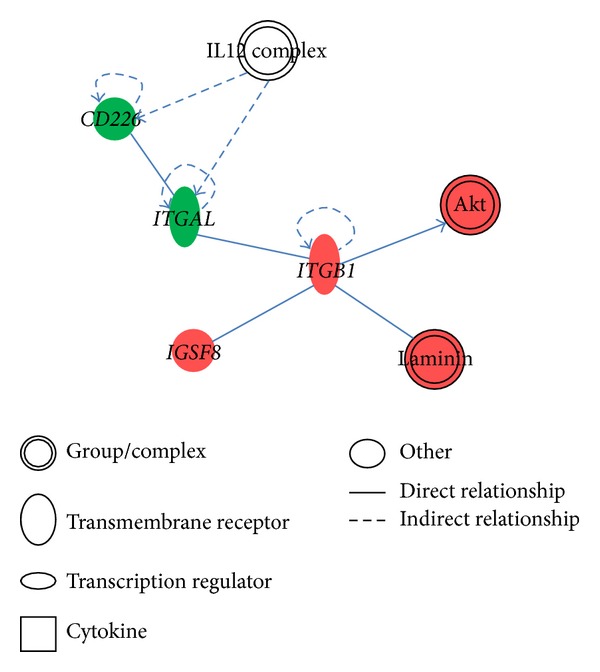
Cellular movement, cancer, tissue development network.

**Figure 3:**
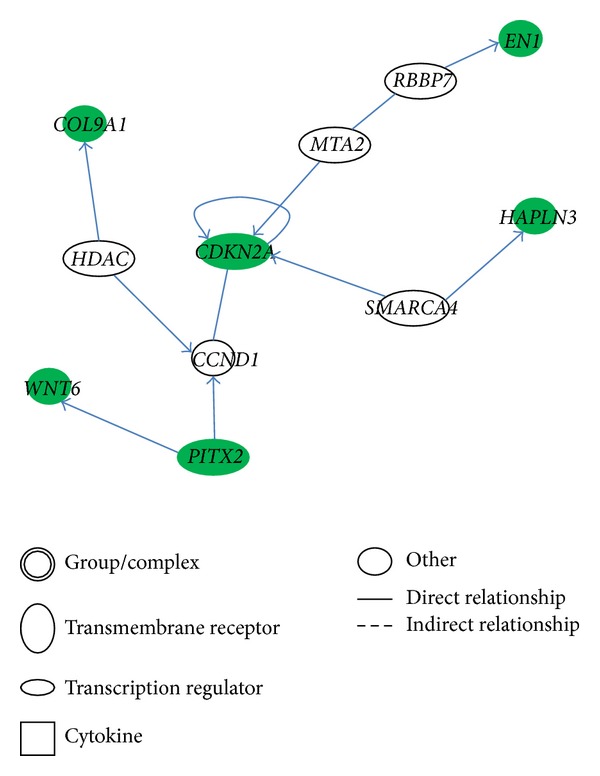
Cell cycle, DNA replication, recombination, and repair, cellular assembly and organization network.

We further explored molecular networks of these cell migration-related genes to cluster gene functions. Among the 16 genes found in non-Asian samples, 7 genes were mapped to three different molecular networks (Table 5).* ITGA6, CCL3*, and* ITGAX* were found to be associated with cell death and survival (Figure 1), while* ITGB1*,* ITGAL*, and* CD226* were mapped to the cellular movement, cancer, and tissue development modules (Figure 2). In the Asian samples, 8 out of 9 genes were mapped to 4 different gene networks. Among the 8 genes,* CDKN2A, EN1*, and* PITX2* were associated with the cell cycle (Figure 3), and the remaining genes were related to organ morphology and cellular development.

In non-Asian samples,* CCL3, ITGAX, ITGA6, ITGB1, ITGAL, CCL21*, and* CD226* were used to mine the pathway. The top three pathways we observed were paxillin signaling, granulocyte adhesion and diapedesis, and integrin signaling. Among the 7 genes we used,* ITGB1, ITGA6*, and* ITGAL* all belonged to the above three pathways. For Asian samples,* MIA, PIK3R3, AR, CCR7, CDKN2A, EN1, PITX2*, and* TFPI* were applied to the pathway analysis process. The top three pathways we observed were chronic myeloid leukemia signaling, p53 signaling, and hepatocyte growth factor (HGF) signaling, and* CDKN2A* and* PIK3R3* were common molecules involved in these three pathways (Table 6).

### 3.4. DAVID Database for Mining Common Pathways

During the IPA pathway analysis process, we found that the top three pathways of non-Asian populations differed from those of Asian populations. However, in non-Asian dataset,* CCL3, ITGA6, ITGAL, ITGAX*, and* ITGB1* were commonly involved in the pathways, paxillin signaling, granulocyte adhesion and diapedesis, and integrin signaling, while* CDKN2A* and* PIK3R3* were common molecules involved in the top three pathways which were observed in Asian dataset. Hence, we applied the DAVID online pathway analysis tool to discover if there were common pathways in which these genes were involved. We found that* PIK3R3, ITGB1*,* ITGA6*, and* ITGAL* were all involved in actin cytoskeleton pathway. Although they were located in different routes of the same pathway,* PIK3R3, ITGB1, ITGA6*, and* ITGAL* indirectly interacted with the common downstream gene,* Rac* (see Supplement S1 in Supplementary Material available online at http://dx.doi.org/10.1155/2014/536591).

## 4. Discussion

The primary goal of this study was to discover common molecules or pathways that affected TNBC cell migration in different ethnic groups. We observed that regulation of the actin cytoskeleton could be one of the most important pathways to affect TNBC cell migration.

Through the SAM calculation process,* ACTA1, ASPN, C4orf7, CYP26B1*, and* PRAME* showed statistically significant differences in expressions between early and late stages of cancer in both Asian and non-Asian samples. Our results showed that* ASPN* was upregulated and annotated in cancer function. This result was consistent with a previous study by Castellana et al. [24]. Their study reported that* ASPN* is highly upregulated in invasive ductal carcinoma (IDC), possibly associated with invasion, and related to the epithelial-mesenchymal transition. In addition,* PRAME* and* CYP26B1* were found to be related to the retinoic acid signaling pathway [25, 26].* PRAME* was reported to be involved in tumor progression rather than tumor initiation by suppressing retinoic acid signaling which regulates gene expression in melanomas [25]. However, these genes were not related to cell migration in our IPA analysis.

We found 16 migration-related genes in the non-Asian populations and 9 migration-related genes in the Taiwanese dataset.* ITGB1* and* ITGA6* were strongly correlated with cell migration and cell growth. Indeed, Ivanova et al. proposed that the upregulated* ITGA6* promotes breast cancer metastasis [27]. Barkan and Chambers also showed that* ITGB1* is a key regulator in the switch from dormancy to active proliferation of tumor cells [28].* ITGB1* and* ITGA6* are genes belonging to* ITG* family which is a receptor on cell membrane. Previous study has indicated that tellurium compound binds with thiols of cysteine residues within the integrin, resulting in antimetastatic activities in melanoma [29]. Thus, this compound may have potential effects on the inhibition of TNBC cell migration.

We found three pathways in non-Asian samples which might play important roles in TNBC cell migration: paxillin signaling, granulocyte adhesion and diapedesis, and integrin signaling. Paxillin is a focal adhesion-associated protein which has been found to be involved in cell migration [30], while integrin was identified to be involved in cell migration, invasion, intravasation, and extravasation, and platelet interactions [31]. Thus, we speculated that TNBC cell migration requires cell-cell and cell-extracellular adhesive forces. In addition, the gene network analysis showed that the immunoglobulin and cytokine* IL12* participated in the top three gene networks (Figures 1 and 2). Therefore, immune system may also participate in TNBC migration. In the Asian dataset, the* CDKN2A* gene with the highest degree of connectivity showed the importance of this molecular in the cell cycle, DNA replication, and recombination network. In addition, a previous study showed that a germline mutation of* CDKN2A* increases the risk of breast cancer [32]. Chronic myeloid leukemia signaling, p53 signaling, and HGF signaling were ranked as the top three pathways. Previous studies demonstrated that higher HGF levels were found in patients with invasive breast cancer compared to controls [33, 34].

By applying the DAVID online analysis tool, we found that* PIK3R3, ITGB1, ITGAL*, and* ITGA6* participated in regulating the actin cytoskeleton (Supplement S1) which supports our hypothesis that a common pathway is involved in TNBC cell migration. Also, these genes had a common downstream gene,* Rac*, which might be a potential target molecule for developing novel treatments for TNBC. However, there are racial differences existing in the cell migration mechanisms. The cell migration mechanism of non-Asian samples was through transmembrane receptor integrin or cell adhesion molecule such as paxillin, while in Asian samples the mechanism of cell migration majorly involved chemotactic and growth factor. These two points may be useful for further study and optimal treatment in the future.

In this study, we used PubMed database and IPA upstream regulator analysis to identify target gene's upstream regulators and observed some miRNAs being potential to regulate the gene expression. For the upstream regulator analysis, Fisher's exact test was applied to calculate the *P* value and *z*-score of expected causal effects between upstream regulators and target genes; the expected causal effects are derived from the literature compiled in the Ingenuity Knowledge Base [35]. We observed that miR-7 is a regulator of* PIK3R3* (Supplement S2), and Xu et al. [36] also indicated that miR-7 seed region matches to the sequence of* PIK3R3* 3′ UTR;thus* PIK3R3* is believed to be a novel target of miR-7. Kong et al. [37] also proposed that miR-7 can inhibit metastasis of breast cancer cells. Additionally,* ITGB1* was predicted to be inhibited by miR-8 (Supplement  2), which has homology to Human-miR-200 family miRNAs [38]. Up to date, there is no record showing that miR-8 expresses in human body. Thus, we suspect that IPA has some defects existing in the database. Nevertheless, because miR-200b and miR-200c are homologous with miR-8, it could be speculated that miR-200b and miR-200c might be the regulators of* ITGB1*. To sum up, miRNA is an important regulator that inhibits target genes and therefore controls the cell migration ability. The computational analysis provides an efficient way to screen through huge amounts of molecular for selection of candidate regulators. However, the results of computational analysis still require further biological studies for validations.

The poor tumor prognosis is the consequence of cancer cell proliferation and migration [39]. However, Kuo et al. (GSE33926) focused on discussing cell proliferation, while we emphasized on studying the tumor cell migration. The combination of these two results may promote the accuracy of TNBC prognosis.

There are limitations in our study. First, data collection was from small sample size. Second, two datasets were generated from different microarray platforms; thus, the number of genes and the sensitivity of platforms might be varied. Further study in a large scale sample size is needed.

In summary,* ITGA6, ITGAL, ITGB1*, and* PIK3R3* were found as important regulators in actin cytoskeleton pathway. Thus, actin cytoskeleton pathways should play important roles in TNBC cell migration. Moreover,* ITGA6, ITGAL, ITGB1, ITGAX, CCL3, PIK3R3*, and* CDKN2A* were found to be important for TNBC cell migration. The associations between these 7 genes and TNBC patients' clinical outcomes require further investigation.

## Supplementary Material

Supplement 1. KEGG pathway analysis indicated that PIK3R3, ITGB1, ITGA6, and ITGAL were involved in actin cytoskeleton pathway.
Supplement 2. miR upstream regulators were identified by IPA analysis.

## Figures and Tables

**Table 1:** Demographic data of patients in the GSE46581 dataset (non-Asian).

	Patients in the early stage (*N* = 25)		Patients in the late stage (*N* = 16)		*P* value
	*n *	%	*n *	%	
Age (years)					
≤39	3	12.00	4	25.00	
40~49	6	24.00	6	37.50	
≥50	16	64.00	5	31.25	**0.1451**
Missing value	0		1	6.25	
Lymph nodes					
Negative	16	64.00	4	25.00	
Positive	2	8.00	7	43.75	<0.05*
Not applicable	7	28.00	5	31.25	

*The statistical significance (*P < 0.05) of the difference between early-stage and late-stage patients was determined by Chi-square test.

**Table 2:** Demographic data of patients in the GSE33926 dataset (Asian).

	Patients in the early stage (*N* = 12)		Patients in the late stage (*N* = 14)		*P* value
	*n*	%	*n*	%	
Age (years)					
≤39	1	8.33	1	7.14	
40~49	2	16.67	4	28.57	
≥50	9	75.00	9	64.29	**0.8555**
Lymph nodes					
Negative	12	100	1	7.14	
Positive	0	0	13	92.85	<0.05*

*The statistical significance (*P < 0.05) of the difference between early-stage and late-stage patients was determined by Chi-square test.

**Table 3:** Common genes between Asian and non-Asian populations.

Gene symbol	Gene description
Up-regulated gene	
*ASPN *	Asporin

Down-regulated genes	
*ACTA1 *	Actin, alpha 1, skeletal muscle
*C4orf7 *	Follicular dendritic cell secreted protein
*CYP26B1 *	CytochromeP450, family26, subfamilyB, polypeptide1
*PRAME *	Preferentially expressed antigen in melanoma

**Table 4:** 25 migration related genes in Asian and non-Asian populations.

	Gene symbol	Multiples of change	Gene description
** Up-regulated genes**			
GSE46581	*ITGB1 *	2.101	Integrin, beta 1
	*RGS1 *	2.231	Regulator of G-protein signaling 1
	*AKT3 *	2.371	v-akt murine thymoma viral oncogene homolog 3
	*ETV4 *	2.103	Ets variant 4
	*HTATIP2 *	2.059	HIV-1 Tat interactive protein 2, 30 kDa
	*IGF1 *	2.231	Insulin-like growth factor 1
	*ITGA6 *	2.295	Integrin, alpha 6
	*LEP *	2.064	Leptin
	*SEMA4D *	2.056	Sema domain, immunoglobulin (Ig domain), transmembrane domain and short cytoplasmic domain, (1emaphoring) 4D

GSE33926	*PIK3R3 *	2.006	Phosphoinositide-3-kinase, regulatory subunit 3 (gamma)
	*AR *	2.106	Androgen receptor
	*CLGN *	2.004	Calmegin
	*TFPI *	2.101	Tissue factor pathway inhibitor

**Down-regulated genes**			
GSE46581	*CCL21 *	0.433	Chemokine (C-C motif) ligand 21
	*CCL3 *	0.389	Chemokine (C-C motif) ligand 3
	*CAMP *	0.464	Cathelicidin antimicrobial peptide
	*CD226 *	0.477	CD226 molecule
	*ITGAL *	0.478	Integrin, alpha L (antigen CD11A (p180), lymphocyte function-associated antigen 1; alpha polypeptide)
	*ITGAX *	0.448	Integrin, alpha X
	*TNFRSF *	0.368	Receptor- (TNFRSF-) interacting serine-threonine kinase 1

GSE33926	*MIA *	0.248	Melanoma inhibitory activity
	*CDKN2A *	0.386	Cyclin-dependent kinase inhibitor 2A
	*EN1 *	0.460	Engrailed homeobox 1
	*PITX2 *	0.476	Paired-like homeodomain 2
	*CCR7 *	0.419	Chemokine (C-C motif) receptor 7

**Table 5:** Migration related gene network in Asian and non-Asian populations.

	Network	Genes
GSE46581	Inflammatory response, cell signaling, cellular movement	*CCL21 *
	Cell death and survival, developmental disorders, skeletal and muscular disorders	*ITGA6*, *CCL3*, *ITGAX *
	Cellular movement, cancer, tissue development	*ITGB1*, *ITGAL*, *CD226 *

GSE33926	Dermatological diseases and conditions, cellular development, cellular growth and proliferation	*MIA*, *PIK3R3 *
	Organ morphology, reproductive system development and function, developmental disorders	*AR*, *CCR7 *
	Cell cycle, DNA replication, recombination, and repair, cellular assembly and organization	*CDKN2A*, *EN1*, *PITX2 *
	Cellular development, cellular growth and proliferation, tumor morphology	*TFPI *

**Table 6:** Migration related canonical pathway in Asian and non-Asian populations.

	Pathway	Genes
GSE46581	Paxillin signaling	*ITGA6*, *ITGAL*, *ITGAX*, *ITGB1 *
	Granulocyte adhesion and diapedesis	*CCL3*, *ITGA6*, *ITGAL*, *ITGB1 *
	Integrin signaling	*ITGA6*, *ITGAL*, *ITGAX*, *ITGB1 *

GSE33926	Chronic myeloid leukemia signaling	*CDKN2A*, *PIK3R3 *
	p53 signaling	*CDKN2A*, *PIK3R3 *
	HGF signaling	*CDKN2A*, *PIK3R3 *
